# Designing and Validation of a Droplet Digital PCR Procedure for Diagnosis and Accurate Quantification of Nervous Necrosis Virus in the Mediterranean Area

**DOI:** 10.3390/pathogens12091155

**Published:** 2023-09-12

**Authors:** Sandra Souto, José G. Olveira, Carmen López-Vázquez, Isabel Bandín, Carlos P. Dopazo

**Affiliations:** Instituto de Acuicultura, Department of Microbiology, Universidade de Santiago de Compostela, 15782 Santiago de Compostela, Spain; sandra.souto@usc.es (S.S.); jose.olveira@usc.es (J.G.O.); mdelcarmen.lopez.vazquez@usc.es (C.L.-V.); isabel.bandin@usc.es (I.B.)

**Keywords:** VNNV, diagnosis, validation, dPCR, droplet digital PCR

## Abstract

The viral nervous necrosis virus (VNNV) is the causative agent of an important disease affecting fish species cultured worldwide. Early and accurate diagnosis is, at present, the most effective control and prevention tool, and molecular techniques have been strongly introduced and accepted by official organizations. Among those, real-time quantitative polymerase chain reaction (rt-qPCR) is nowadays displacing other molecular techniques. However, another PCR-based technology, droplet digital PCR (ddPCR), is on the increase. It has many advantages over qPCR, such as higher sensitivity and more reliability of the quantification. Therefore, we decided to design and validate a protocol for the diagnosis and quantification of SJ and RG type VNNV using reverse transcription-ddPCR (RT-ddPCR). We obtained an extremely low limit of detection, 10- to 100-fold lower than with RT-qPCR. Quantification by RT-ddPCR, with a dynamic range of 6.8–6.8 × 10^4^ (SJ type) or 1.04 × 10^1^–1.04 × 10^5^ (RG type) cps/rctn, was more reliable than with RT-qPCR. The procedure was tested and validated in field samples, providing high clinical sensitivity and negative predictive values. In conclusion, we propose this method to substitute RT-qPCR protocols because it exceeds the expectations of qPCR in the diagnosis and quantification of VNNV.

## 1. Introduction

Real-time quantitative polymerase chain reaction (rt-qPCR) is nowadays a reference for the detection and quantification of fish viruses, including the viral nervous necrosis virus (VNNV). These viruses, corresponding to the genus *Betanodavirus* within the family *Nodaviridae*, produce a neurological disease with serious consequences on certain species of worldwide farmed fish [1]. They are small (25–30 nm), unenveloped, single-stranded positive-sense RNA viruses whose genome is bisegmented. The largest segment (RNA 1) codifies the RNA-dependent RNA-polymerase, and the other one (RNA 2) codifies the capsid protein.

Traditionally, four genotypes have been considered, named after the original host species: striped jack nervous necrosis virus (SJNNV), red-spotted grouper NNV (RGNNV), tiger puffer NNV (TPNNV), and burfin flounder NNV (BFNNV). In addition to this basic classification, some authors have proposed the inclusion of subgroups within the BF and SJ types, depending on the geographic origin of the isolate. A fifth genotype, detected in turbot (Turbot nodavirus, TNV), is widely accepted, although no isolates are available. Moreover, the inclusion of two additional genotypes has been proposed: the atlantic cod nervous necrosis virus (ACNNV), which is now considered a clade within the BF type, and more recently, the korean shellfish NNV (KSNNV), for which there is not enough information available to support its consideration as a new genotype [1].

Due to the importance of the disease in the Mediterranean area, where its impact on aquaculture represents a great concern for fish farmers, the detection of the agent in early phases and the selection of non-carrier broodstock is extremely important. Therefore, it is necessary to develop increasingly faster, more sensitive and reliable diagnostic methods. The World Organization of Animal Health (WOAH) has recently removed this virus from its list of risk aquatic viruses. However, in a previous version of its Manual of Diagnostic Test of Aquatic Diseases [2], they considered that, together with the traditional gold standard diagnostic test (isolation in cell culture followed by immunological or molecular confirmation), highly sensitive molecular techniques could be employed but require previous validation. They placed special emphasis on the use of certain real-time PCR (rtPCR) protocols and mainly focused on the only truly validated technique (including a ring test) at that time, the one developed by the OIE Reference Laboratory for Viral Encephalopathy and Retinopathy [3].

More recently, Olveira et al. [4] reported the development and validation of a procedure not only for the detection and identification but also for the quantification of all types of viruses in cell culture supernatants and in fish tissues. In addition to the high sensitivity of that protocol (higher than any precursor), another advantage was that, after validation, quantification was demonstrated to be highly reliable with any of the standards used, which allows for the comparison between different laboratories.

With a view to improving our capacity to detect this virus in field samples and for its precise quantification, we explored a related emerging technology, the droplet digital PCR (ddPCR), which is widely used for the early detection of cancer [5,6] and human viruses [7,8]. Digital PCR (dPCR) is a novel and promising technology that will probably replace qPCR due to its advantages. Actually, it is not that new because it was first described in the late 1980s as a method for the detection of a single nucleic acid molecule and in the early 1990s for the quantification of viruses [9]. However, the term ‘digital PCR’ was introduced in the late 1990s.

The advantages of dPCR over qPCR have been described [7,10]: (i) higher sensitivity due to the template concentration effect of the partitioning; (ii) direct absolute nucleic acid quantification without the need to use standard curves and with higher precision; (iii) less susceptibility to impaired efficiencies due to PCR inhibitors. The technology is based on the distribution of the templates into microscopical partitions, following a Poisson distribution, and on PCR amplifications individually performed within each partition. There are two types of methods, depending on the partitioning system: the chip-based method, where the partitions are solid microcells, and the oil-emulsion partitions, called droplets, corresponding to the ddPCR technology.

Based on the reported advantages of ddPCR over chip-based PCR due to the larger number of partitions involved, which provides a larger dynamic range [10], we chose ddPCR to develop a new protocol to improve our capability to detect, identify and quantify VNNV isolated in cell culture, and directly from fish tissues. The new procedure has been demonstrated to be more sensitive and reliable for the detection and quantification of this virus, which confirms it as a future substitute for qPCR, improving our capabilities to control this disease.

## 2. Materials and Methods

### 2.1. Viral Strains, Cell Culture and Viral Titration

For reference, in the present study, we selected the SJNag93 and SGWAK97 VNNV strains, corresponding to types SJNNV and RGNNV (respectively); the two VNNV types are most frequently detected in Mediterranean countries and worldwide. For their propagation, E-11 cells (ECACC #01110916) were employed using L-15 (Leibovitz; Lonza, Vigo, Spain) culture medium supplemented with 5% fetal bovine serum (FBS; Lonza). Cell culture was carried out at 25 °C, and inoculated cells were incubated at 20 °C. After extensive cytopathic effect (CPE), cell debris from the culture fluids was removed at 3000× *g* for 15 min at 4 °C. Viral titration was performed in 96-well plates using the endpoint dilution method as described by [4].

### 2.2. Optimization of the Procedure

Since the aim of this study was to compare the performance and reliability of the designed RT-ddPCR procedure against the RT-qPCR test routinely employed in our laboratory, the latter was used as a reference. This RT-qPCR protocol is based on a study partially published in 2021 by Olveira et al. [4], where many more primer sets than reported had been tested, and some probes were also evaluated. In that study, an in silico test was applied on the reference strains from the four main genotypes (SGWak97 (RG type), SJ93Nag (SJ type), JFIWa98 (BF type) and TPKag93 (TP type)), testing putative primers/probe (Pr/Pb) sets in both RNA segments (Accession numbers for RNA1/RNA2: NC_008040/NC_008041, AB056571/AB056572, NC_013458/NC_013459, and NC_013460/NC_013461, respectively). In silico, the best options (appropriate for the four types) were sets located in the RNA1 segment, and that pair of primers was the one validated in that report because it provided the best results for the detection and quantification of the four VNNV type strains (SJ, RG, TP and BF). Another set of primers and a probe (Pr/Pb), specific for RNA 2, had also been tested, providing optimal results only with SJ and RG types. Those results were not published; however, the Pr/Pb set is the one used in the RT-qPCR procedure we routinely use for diagnosis and quantification of VNNV in isolated virus and field samples, given that SJ and RG types have been demonstrated for years to be the only types detected in the samples received from companies in the Mediterranean area.

In the present study, said Pr/Pb set (Table 1) was used for both RT-qPCR and RT-ddPCR and the concentrations and the annealing temperatures were the parameters optimized for ddPCR. To this end, Pr/Pb concentrations of 500/250, 500/500, 750/250, 750/500, 950/500 and 950/750 nM were tested at an annealing temperature of 57 °C (the reference from the routine RT-qPCR procedure). In another set of tests, six annealing temperatures (52, 54, 56, 57, 58 and 60 °C) were tested using the Pr/Pb set at 500/500 nM.

After the ddPCR amplification, the optimal parameters were selected considering the amplitude (separation between negative and positive droplet populations), the rain (droplets falling between both populations), and the repeatability of the counting of the reference templates.

### 2.3. Reference Templates

For the evaluation of the RT-ddPCR procedure and for comparison with RT-qPCR, two types of templates were used: 

Titrated crude virus and extracted RNA.- SJ and RG strains were produced and titrated as described above, and viral RNA was extracted using the RNeasy Mini Kit (Qiagen, Madrid, Spain) following the procedure described by the manufacturer. Quantification and quality of the RNA were determined as described by Olveira et al. [4].

Plasmid DNA (pDNA).- For each strain, a different pDNA was designed and constructed as described by Olveira et al. (2021): plasmid PGEMT, with an insert of 1421 bp corresponding to RNA 2 of SJNNV (total length 3990 bp), and plasmid BPST7, with an insert of 1430 bp, corresponding to RNA 2 of RGNNV (total length 4435 bp).

### 2.4. cDNA Synthesis

For amplification by qPCR and ddPCR, cDNA was synthesized as previously described [4]. Briefly: 9 µL of extracted RNA (around 1 ng/µL) were mixed with 2.5 ng/µL of random primers (random hexadeoxynucleotides; Promega, Madrid, Spain), and then subjected to 95 °C, 5 min, and 4 °C for more than 1 min. For reverse transcription (RT), the Superscript III reverse transcriptase (10 U/mL; Invitrogen, Madrid, Spain), 0.5 mM dNTPs and 0.05 M DTT in First Strand buffer (Invitrogen) were added to a final volume of 20 µL and incubated 10 min at 25 °C and then 50 min at 50 °C, in a My Cycler thermal cycler (Bio-Rad, Madrid, Spain). Immediately after cDNA synthesis, the RT enzyme was inactivated at 85 °C for 5 min, and the cDNA samples were conserved at −20 °C until use.

### 2.5. Real-Time Quantitative PCR (rt-qPCR)

For the rt-qPCR reaction, a mixture containing 2 µL of cDNA, 500 nM of each primer and a probe in PremixTM Ex Taq (Takara bio INC, Shiga, Japan) was prepared to a final volume of 20 µL. For the amplification, after an initial 30 s denaturation/activation step at 95 °C, 45 amplification cycles were applied in a CFX Connect™ Thermal Cycler (Bio-Rad, Madrid, Spain) as follows: denaturation for 15 s at 95 °C; annealing and extension for 20 s at 57 °C. For each run, the threshold cycle value (Ct) was established as the cycle number at which fluorescence was detectable over the threshold value determined by the equipment’s software (CFX Maestro; Bio-Rad) for cycles 2–10.

### 2.6. Droplet Digital PCR (ddPCR)

The ddPCR reactions were carried out into a final volume of 20 µL containing 500 nM of primers and probe and 2 µL of cDNA in ddPCRTM Supermix for Probes (No dUTP) (Bio-Rad). A QX200TM Droplet Generator (Bio-Rad) was used to generate the partitions. Following an initial 10 min denaturation/activation step at 95 °C, the mixture was subjected to 40 cycles of amplification (20 s denaturation at 95 °C, followed by 30 s at 58 °C of annealing and extension) in a C1000TM thermal cycler (Bio-Rad), and the droplets were individually read using a QX200TM Droplet Reader (Bio-Rad). Only reactions with a total number of droplets higher than 10,000 were considered. The results were analyzed by Quanta SoftTM Pro vs. 1.0.

### 2.7. Limit of Detection, Quantification and Dynamic Range

Serial dilutions (10-fold) of reference templates were subjected to amplification by RT-qPCR or RT-ddPCR when using viral RNA as a template and qPCR or ddPCR when pDNA was employed.

Crude virus. Crude virus stocks of 5.6 × 10^7^ TCID_50_/mL and 1.0 × 10^6^ TCID_50_/mL of SJNNV and RGNNV strains, respectively, were employed. Dilutions from 0 to 10^−10^ were used to determine the limits of detection (LOD) and quantification (LOQ) in terms of viral concentration. To determine the LOD and LOQ values in terms of viral copies, RNA extraction was applied from 100 µL of crude virus, and the RNA concentration was measured as described above. The number of copies was calculated from the formula **γ** = n/N × GL × ncMW, where **γ** is the RNA weight in gr, n is the number of copies, N the Avogadro number (6.022 × 1023 copies/mol), GL the total genome length (4528 and 4539 nc, for SJ and RG types, respectively), and ncMW is the nucleic acid molecular weight (average values: 350.5 and 328.0 gr/mol, SJ and RG, respectively).

To determine the viral titer and number of copies per reaction, the procedure followed to obtain the two microliters of cDNA for the ddPCR mix was taken into account: RNA extracted from 100 µL of crude virus was concentrated into a volume of 70 µL from which 9 µL were used for cDNA synthesis to a final volume of 20 µL, from which 2 µL were used for ddPCR to a final volume of 20 µL.

pDNA. In the case of using pDNA as a reference template, 45 ng/µL or 23 ng/µL stock solutions (SJ and RG, respectively) were employed, and 2 µL was used for the ddPCR 20 µL total reaction volume. To calculate the number of copies, the same formula for **γ** was employed, using a ncMW average value of 620 or 660 gr/mL (SJ and RG, respectively) and the plasmid sizes described above.

To determine the LOD, the highest dilution providing positive results in a minimum of 1/3 of the replicas and with an acceptable CV value (CV ≤ 25%) was considered. For the LOQ, the lowest concentration within the dynamic range (DR) was contemplated. The DR was determined as the range of dilutions (or concentrations) providing reliable quantification (correlation coefficient of the curves (R2) ≥ 0.95).

### 2.8. Reliability of the Procedure

Repeatability and reproducibility (R and R) were measured to evaluate the procedure’s reliability. For that purpose, each assay was performed with a minimum of three replicas (repeatability) and on consecutive days (reproducibility). R and R were determined from the magnitude of the deviation from the averaged template concentrations, using the ‘coefficient of variation’ (CV; or RSD, ‘relative standard deviation’), calculated as the percentage of standard deviation with respect to the average. Values ≤ 25% were indicative of acceptable reliability [11].

In addition, standard curves were drawn with the data obtained using the serial dilutions of the reference templates, and their reliability was determined from the calculated correlation coefficient (R2). Curves with R2 ≥ 0.95 were considered reliable.

### 2.9. Performance of the Procedure with Field Samples

The procedure was tested on fish samples in collaboration with a *Solea senegalensis* fish farm in the Iberian Peninsula as part of a program to select VNNV-free breeders. The sampling was performed on the fish farm by their technicians, with their own approved protocols, and under their responsibility. Briefly: Twenty-four fish were anesthetized with MS-222 (Tricaine Methanesulfonate) prior to puncturing the caudal vein, and the blood samples (maximum volume 500 µL) immediately heparinized. The blood samples were sent at 10 °C to our laboratory within 24 h. Nucleic acid extraction was performed as previously described [12].

To evaluate the clinical reliability of the diagnostic test, the following parameters were calculated, as described [13], always in reference to a gold standard test:

Clinical sensitivity (cSs). Calculated as the relation (in terms of frequency or percentage) between the observed (with the test under evaluation) and the expected (with the gold standard) positive results. The higher the frequency (maximum value 1), the higher the cSs, defined as the capacity to detect diseased fish, and this is directly related to the analytical Ss.

Clinical specificity (cSp). This is a different concept from analytical Sp since it is not related to the capacity to detect any viral type and no other viral groups but to the capacity to detect true uninfected fish (the need to avoid false negatives). This parameter is calculated from the relation between the number of negatives obtained with the evaluated test and the total expected from the gold standard.

Positive predictive value (PPV). This parameter provides a measurement of the reliability of the positive results and is calculated as the relation between the observed true positives (results that are positive with both tests) and the total number of positives with the test under evaluation.

Negative predictive value (NPV), which gives the reliability of the negative results, is calculated from the relation between true negatives (results that are negative with both tests) and the total number of negatives with the evaluated test.

In all four cases, the higher the value (1 or 100, frequency or percentage, respectively), the better the procedure. In the present study, two approaches were contemplated: using qPCR and ddPCR, alternatively, as a gold standard.

### 2.10. Statistical Analysis

To compare the quantification data obtained by both procedures (qPCR and ddPCR), three tests were consecutively applied using Prism vs. 10.0 (GraphPad): a Student’s *t*-test, an unpaired *t*-test with Welch’s correction (not assuming equal SDs), and a Wilcoxon test. Values of *p* ≤ 0.05 mean significative differences between results.

## 3. Results

### 3.1. Optimization of the Conditions

**Figure 1:**
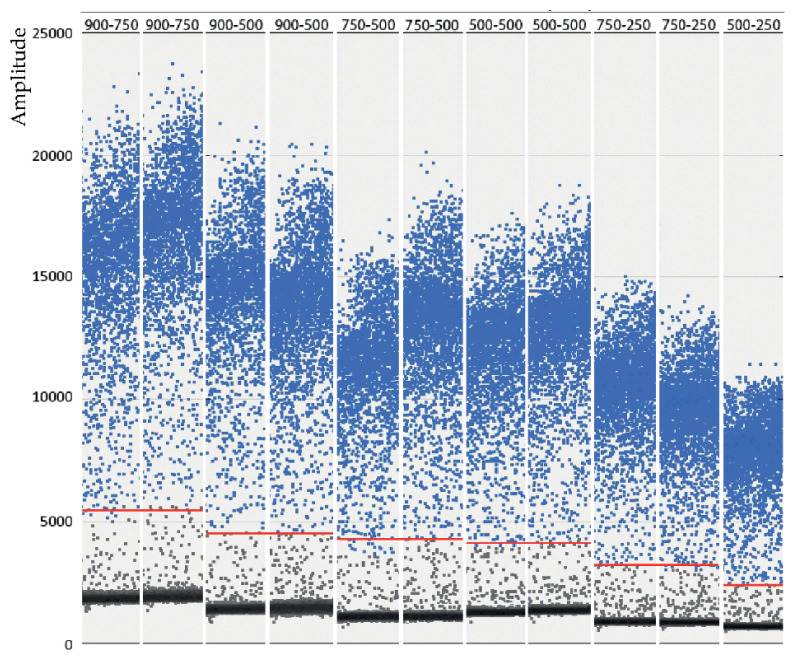
Optimization of the primers and probe concentration: Amplitude and rain. Several combinations of primers and probe concentrations (from 500–250 to 900–750 nM [primers-probe, respectively]) have been tested analyzing the amplitude (separation between negative and positive droplets populations), and the rain (droplets failing between both populations). The red line shows the threshold between negative and positive droplets.

**Figure 2:**
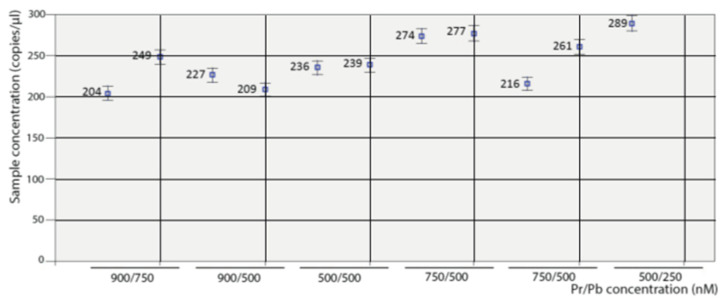
Optimization of the primers and probe concentration: Repeatability.

Optimization of the conditions was performed by testing six annealing temperatures, from 52 to 60 °C, and six combinations of Pr/Pb concentrations, from 500/250 to 900/750 nM. As shown in the scatter plot of Figure 1, the maximum amplitude was obtained with the highest concentration of primers (900 nM) at both probe concentrations tested (500 and 750 nM). Enough separation between the two droplet populations (positive and negative) and with a similar level of rain was also observed at the 500/500 nM combination. With the remaining concentrations, the amplitude was too low and/or the rain was excessive. Based on these results, the logical choice would be either of the first two combinations (900/500 or 900/750); however, as shown in Figure 2, the results (using two replicas) were not repeatable at those concentrations. We must point out that deviations due to variations in the number of readable droplets between repeats or due to low numbers of dots were discarded (Figure S1). Therefore, the combination 500/500 nM was chosen for the concentration of primers and probes in the ddPCR procedure.

**Figure 3:**
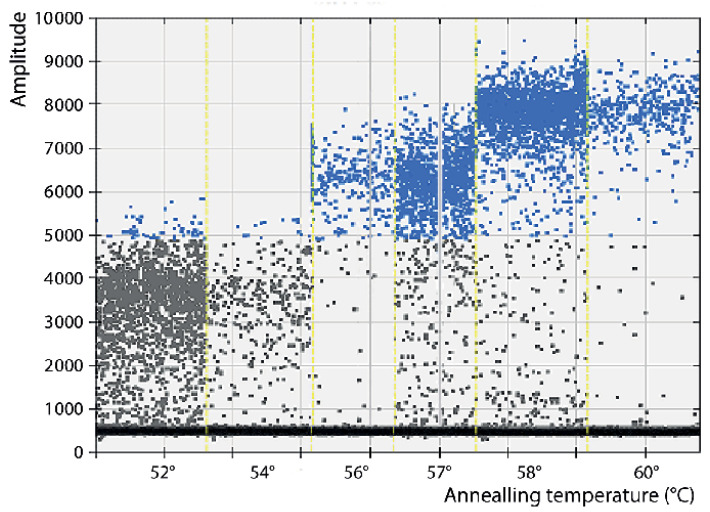
Optimization of the Annealing temperature: Amplitude and rain. Annealing temperatures (separated by yellow dashed lines) between 52 and 60°C have been tested, analyzing the amplitude (separation between negative and positive droplets populations), and the rain (droplets failing between both populations).

Regarding the annealing temperature (Figure 3), based on the amplitudes, and because they provided quantification values closer to the original template concentration, 57 and 58 °C were the best options. Given that 57 °C was the annealing temperature originally employed in the qPCR protocol, it was also maintained in the present study for those assays involving qPCR. However, because less rain was obtained at 58 °C, this annealing temperature was chosen for the ddPCR runs.

### 3.2. Repeatability, Reproducibility and Specificity

To evaluate repeatability, all the assays were carried out, applying at least three replicas with both reference templates (plasmid and crude virus) and both VNNV types (SJ and RG). There was only one exception, the fourth repeat using SJ type pDNA (Tables S1–S5), in which case only one replica was used as it was carried out to test the highest dilutions.

With the RG type and within the quantification range (see below), all the repeats demonstrated to be repeatable, with CV values ≤ 25%, except in one case, namely SJ crude virus in repeat 2 at dilution 10^−8^, where a CV of 28.3 was obtained (Table S9). For all the remaining, CV data were between 0.3 and 24.95, or 9.91 and 22.65 (SJ and RG types, respectively) with pDNA, and between 2.9 and 24.2 (SJ) or 9.67 and 21.4 (RG) with crude virus (see Tables S1–S11).

To evaluate reproducibility, all the data were set together (see Tables S12–S15), and again, the CV values were maintained ≤ 25%, except for the aforementioned high dilution of SJ crude virus (Table S12), which suggests a certain deviation (not too high: CV = 32.2) at the lowest concentrations with one of the type strains.

When analyzing the results of reproducibility with RT-qPCR, in the RG type, all CV values were below 25% (Tables S14 and S15); with SJ, they were extremely high at all dilutions, from 49.25 to 167.99% (Table S12). As expected, the CV values were reduced below 10 in most cases when the decimal logarithm of the quantification data was used instead of the absolute values.

### 3.3. Dynamic Range (DR); Limit of Detection (LOD) and Limit of Quantification (LOQ)

Both detection and quantification DR were generally lower with ddPCR than with qPCR (Table 2 and Table 3). For quantification, the DR with ddPCR was always of 5 Log_10_, in a range between attograms (ag) and femtograms (fg) with plasmid and ag and picograms (pg) with crude virus. The DR was always higher with qPCR, between 6 and 9 Log_10_, depending on the template and the viral strain. The DR for detection increased up to 2 Log_10_, with the RG type strain, or a maximum of 1, with SJ.

The LOD per reaction (rctn) using pDNA as reference was in the order of 0.9 ag (equivalent to 0.22 copies/rctn) with SJ and 4.6 ag (0.95 cps/rctn) with RG (Table 2 and Table 3), 10 times lower than with qPCR. With the crude virus, the LOD per rctn was 18 ag with SJ and 2.6 ag with RG. This corresponds to 6.8 and 0.1 genome copies/rctn or 7.2 × 10^−4^ and 1.3 × 10^−5^ TCID_50_/rctn (SJ and RG, respectively) and represents a much higher sensitivity than with qPCR (between 1 and 2 Log_10_ lower LOD). It is interesting to note that with the crude virus, there is a 1/10^−4^ ratio between RNA cps/rctn (calculated based on the weight of extracted RNA and the Avogadro formula) and the viral titers/rctn.

### 3.4. Reliability of the Quantification

**Figure 4:**
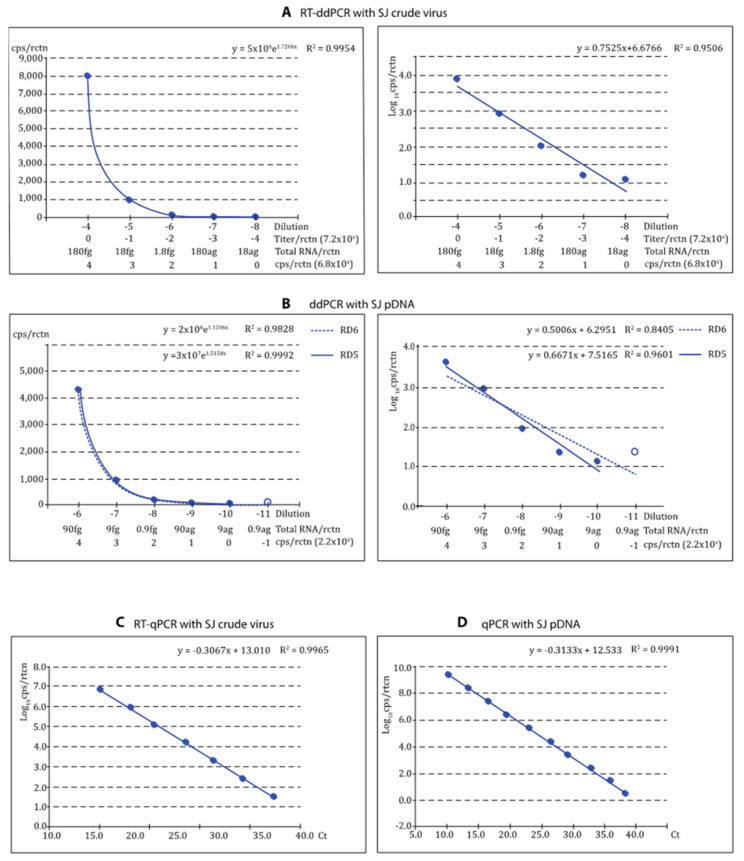
Reliability of the ddPCR procedures for quantification if th SJ type viruses using crude virus (RT-ddPCR) and pDNA (ddPCR) as reference. The reliability of the procedure for quantification of VNNV (SJ-type) was evaluated based on the correlation coefficient values (R^2^) of the regression curves obtained: RT-ddPCR or ddPCR applied to quantify SJNNV crude virus (**A**) or SJ-type pDNA (**B**), respectively; exponential and linear regression curves (left and right, respectively) are shown with dynamic ranges (RD) 5 (curves A) or 5–6 (curves B); for each graph B, the low DR excludes the empty circle data. RT-qPCR with SJ crude virus (**C**) and qPCR with SJ pDNA (**D**) were used as reference.

**Figure 5:**
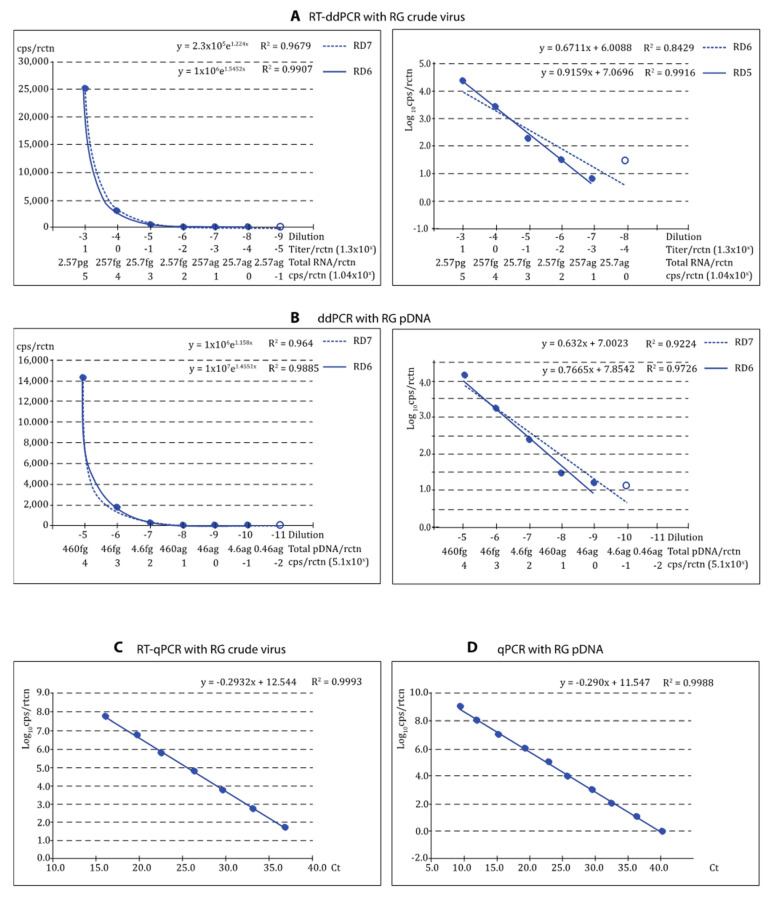
Reliability of the ddPCR procedures for quantification if th RG type viruses using crude virus (RT-ddPCR) and pDNA (ddPCR) as reference. The reliability of the procedure for quantification of VNNV (RG-type) was evaluated based on the correlation coefficient values (R^2^) of the regression curves obtained: RT-ddPCR or ddPCR applied to quantify RGNNV crude virus (**A**) or RG-type pDNA (**B**), respectively; exponential and linear regression curves (left and right, respectively) are shown with dynamic ranges (RD) 6–7 (exponential curves) or 5–6 (linear curves); for each graph, the low DR excludes the empty circle data. RT-qPCR with RG crude virus (**C**) and qPCR with RG pDNA (**D**) were used as reference.

As shown in Figure 4 and Figure 5, the regression curves were highly reliable, with at least the expected DR of 5 Log_10_, with both viral types and reference templates. Using the absolute values, the exponential regression curves showed *p* > 0.99 in all cases, and *p* was at least 0.95 in linear regression when the logarithmic values were used. The curves calculated for each repeat of all tests can be visualized in Figures S2–S5. For qPCR, DR was 7 Log_10_ with crude virus and 10 with pDNA (Figure 4C,D and Figure 5C,D).

### 3.5. Correlation between ddPCR and qPCR Quantification Methods

**Figure 6:**
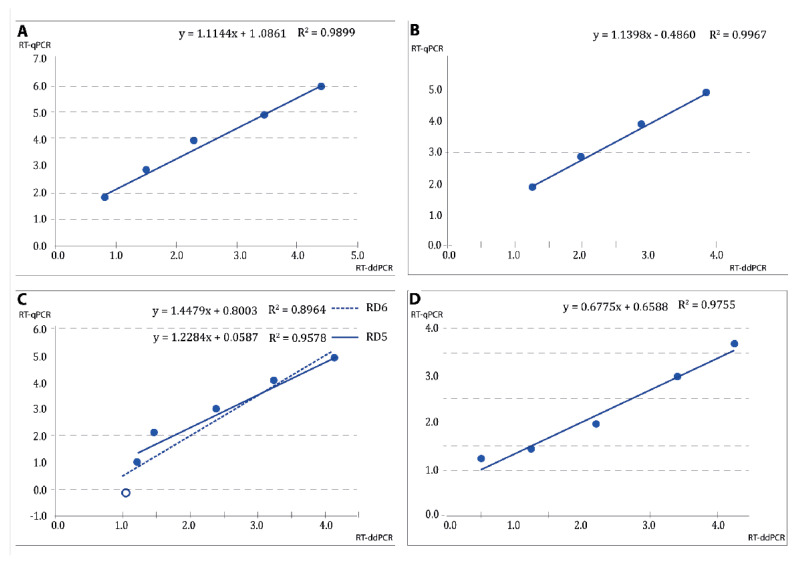
Correlation between qPCR and ddPCR quantification procedures. Correlation between qPCR and ddPCR quantification procedures, in terms of Log10cps/rctn (copies per reaction). (**A**) RNA from crude virus (RG-type); (**B**) RNA from crude virus (SJ-type); (**C**) RG-type pDNA; (**D**) SJ-type pDNA.

Correlation between the measurements obtained with both procedures seemed to be demonstrated by the correlation coefficients in all cases (Figure 6). However, because in many cases, the data looked really different to the naked eye, a Student’s *t*-test was applied, obtaining *p* values always higher than 0.05. Then, we tried the Welch correction with similar results, and finally, the Wilcoxon test was applied, with no differences either.

### 3.6. Performance of the RT-ddPCR Procedure in Field Samples

To validate the procedure for diagnosis, it was tested in field samples of expected extremely low viral loads: blood samples from breeders. It was carried out as part of a program for the selection of VNNV-free breeders, and for which our RT-qPCR protocol is routinely employed. As shown in Table 4, some of the fish considered VNNV-free by RT-qPCR (Ct ≥ 40) were confirmed positive by RT-ddPCR. On the other hand, two cases of Ct > 3.95, considered weak positives by RT-qPCR (but enough to reject the specimens as breeders), are actually virus-free as determined by RT-ddPCR.

To evaluate the clinical parameters, the results with both methods were compared in a contingency table, alternating both methods as the gold standard and comparing the results obtained by changing the threshold Ct for positives (Ct < 40 or ≤39.5). As shown in Table 5, the clinical sensitivity of the ddPCR procedure was extremely high (cSs = 1) when the threshold for positive results was lowered to just 0.5, and the reliability of negatives (NPV) was also maximum.

## 4. Discussion

Detection of the VNNV virus in disease episodes using WOAH-recommended procedures is quite reliable due to the high viral loads present in the tissues of acutely infected fish. In these cases, lethal sampling is used to process certain internal organs. This technique cannot be used to select agent-free breeders. In this case, the blood of the specimens is sampled to apply RT-qPCR to detect the virus in the lymphocytes. Viral loads in asymptomatic carrier breeders are extremely low, which means further difficulty in the detection of the agent. In addition, the reliability of the quantification at the high Ct values obtained makes risk analysis difficult. Therefore, it is necessary to develop procedures and technologies that provide greater sensitivity and greater reliability in these situations.

Digital PCR (dPCR) is an accurate technology for the detection and quantification of nucleic acid templates, which has proven important advantages over rtPCR, such as higher sensitivity and more reliability of quantification, because it does not depend on standard curves [10]. Furthermore, among the dPCR variants, ddPCR outperforms digital chip PCR [14]. For this reason, we chose ddPCR technology to adapt our laboratory to the future of diagnosis of viral pathologies in aquaculture.

Diagnosis by ddPCR has been extensively introduced in human viral diseases and for a wide range of viruses, such as influenza, leukemia, encephalitis, hepatitis (A and B), norovirus, papilloma and polyoma, SARS and Zika [14]. However, few reports on the use of ddPCR for the diagnosis and quantification of fish viruses have been published, and even less have addressed the evaluation (and none the complete validation) of the reported procedure. A first approach by Jia et al. [15] developed and validated (just from an analytical point of view) a ddPCR procedure for the detection and quantification of the infectious haematopoietic necrosis virus (IHNV). They reported a LOD of 2.2 pfu/µL, surprisingly higher than the data they obtained with qPCR. Assuming an equivalence of 4.4 pfu/rctn, it would be much higher than the LOD that we obtained in terms of titer: in the order of 10^−4^ (with SJ) and 10^−5^ (with RG) TCID_50_/rctn. However, as indicated in the Results, we have observed a 1/10^−4^ ratio between copies and crude virus titer. Therefore, the LOD reported by those authors would be between the LOD values we observed with RGNNV (0.1 cps/rctn) and SJNNV (6.8 cps/rctn). Similar LOD values have been reported by other authors for infectious spleen and kidney necrosis virus (3 cps/rctn; [16]), carp edema virus (2.2 cps/rctn; [17]), largemouth bass ranavirus (2.0 cps/rctn; [18]) or tilapia lake virus (3.3 cps/rtcn; [19]). A lower LOD, 0.07 cps/µL, has been reported for a method developed for tilapia parvovirus [20]; assuming a volume of 2 µL of template per ddRNA reaction mixture, that LOD would be equivalent to 0.14 cps/rctn, quite similar to what we obtained for the RG type with our protocol. Similar analytical sensitivities, but also higher LOD values, have been reported by other authors, as reviewed by Lei et al. [14] and Chen et al. [8].

The LOQ is expected to be higher than the LOD; this is because, to accept a dynamic range for quantification, the linearity between the measurements of serially diluted samples usually fails at the lower concentrations, providing acceptable numbers of positive droplets. Moreover, as expected, the LOQ obtained with the RG type strain was 10 times higher (LOQ = 10.4 cps/rctn) than the LOD (0.1 cps/rctn); however, this was not the case with the SJ type, which provided a value of 6.8 cps/rctn for both LOD and LOQ. We must note that the reliability of the quantification was ensured by the linearity of all the curves, with R2 values always >0.95. These values are below those reported by other authors, such as Han et al. [21], who reported a LOQ of 20 or 25 cps/rctn, depending on the virus (hepatitis A or norovirus g.I or g.II), or Mairiang et al. [22], who obtained a LOQ of 2.337, but in terms of Loq_10_cps/rctn, actually corresponds to 217 cps/rctn.

LOQ and LOD define the lowest limit of the DR, but in ddPCR, it clearly depends on the saturation of positive droplets, which makes the Poisson distribution invalid at template concentrations over 10^5^ [17,23,24]. Therefore, the 5 Log_10_ DR values observed in the present study were within those expected and reported in most cases, although others have been able to reach DRs of 6 Log_10_ [25,26].

Although this limit in DR looks like a disadvantage of ddPCR with respect to qPCR (which can easily reach DR values of 10 Log_10_) because failures can occur if highly concentrated viruses are measured, it is compensated by its higher precision [10]. In this regard, the evaluation of repeatability and reproducibility of the RT-ddPCR developed here has provided CV values within the limit of 25% for both VNNV types. On the contrary, the deviations reached with RT-qPCR were extremely high with one of the viral types. We must mention that this is not unusual, although rarely shown in qPCR validation procedures, given that R and R are always evaluated in terms of Ct values, and not template quantities.

To ensure the validation of a procedure for the diagnosis of fish viral diseases, the analytical point of view is only a first step, and the clinical –or diagnostic– approach is also mandatory [27]. Therefore, we chose a type of sample that is extremely complicated due to the type of tissue itself (blood) and also due to the expected viral load (very low since it is from asymptomatic breeder fish). The designed RT-ddPCR procedure has been demonstrated to be more reliable than RT-qPCR for detecting VNNV in field samples because it provided higher clinical sensitivity and NPV values, reaching the maximum when false positives by RT-qPCR were avoided by reducing the threshold Ct for positive reactions.

## 5. Conclusions

In conclusion, we have designed and formally validated an RT-ddPCR procedure for the diagnosis and quantification of the nervous necrosis virus VNNV. The procedure has demonstrated higher reliability and performance than RT-qPCR and represents the first protocol available for the control of an important disease in Europe and worldwide.

## Figures and Tables

**Table 1:** Primers and probe.

Pr/Pb ^1^	Name	S/As ^2^	Sequence (5′ to 3′)	Position ^3^	Ampl. Size ^4^
Pr	T_NodR2_330	S	TACGCTGTTGAAACACTG	330–347	100 bp
Pr	T_NodR2_430	As	CGTTGTCAGTTGGATCAG	429–412	
Pb	TQM_NodR2_359	S	ATTCAGCCAATGTG	357–370	

^1^ Pr/Pb: Primers and Probe; ^2^ S/As: Sense or antisense; ^3^ Position: nucleotide positions in the strain SGwak97 RNA 2 (NC_008041); ^4^ Ampl. Size: amplicon size in base pairs.

**Table 2:** Dynamic range; Limit of Detection and Limit of Quantification with SJ type VNNV.

	Limit of Detection				Dynamic Range for Detection			
	Plasmid		Crude Virus		Plasmid		Crude Virus	
	qPCR	ddPCR	qPCR	ddPCR	qPCR	ddPCR	qPCR	ddPCR
Dilution ^1^	−10	−11	−7	−8	−2 to −10	−6 to −11	−2 to −7	−4 to −8
w/reaction ^2^	9 ag	0.9 ag	0.18 fg	18 ag	0.9 ng–9 ag	90 fg–0.9 ag	18 pg–0.18 fg	0.18 pg–18 ag
cps/reaction ^3^	2.2	2.2 × 10^−1^	68	6.8	2.19 × 10^8^–2.19	2.19 × 10^4^–2.19 × 10^−1^	6.8 × 10^6^–6.8 × 10^1^	6.8 × 10^4^–6.8 × 10^0^
Tit/react ^4^	N/A	N/A	7.2 × 10^−3^	7.2 × 10^−4^	N/A	N/A	7.2 × 10^2^–7.2 × 10^−3^	7.2 × 10^0^–7.2 × 10^−4^
Tit/mL ^5^	N/A	N/A	5.6	0.56	N/A	N/A	5.6 × 10^5^–5.6 × 10^0^	5.6 × 10^3^–0.56
	**Limit of Quantification**				**Dynamic range for quantification**			
	**Plasmid**		**Crude virus**		**Plasmid**		**Crude virus**	
	**qPCR**	**ddPCR**	**qPCR**	**ddPCR**	**qPCR**	**ddPCR**	**qPCR**	**ddPCR**
Dilution ^1^	−9	−10	−7	−8	−2 to −9	−6 to −10	−2 to −7	−4 to −8
w/reaction ^2^	90 ag	9 ag	0.18 fg	18 ag	0.9 ng–90 ag	90 fg–9 ag	18 pg–0.18 fg	0.18–pg 18 ag
cps/reaction ^3^	21.9	2.19	68	6.8	2.19 × 10^8^–21.9	2.19 × 10^4^–2.19	6.8 × 10^6^–6.8 × 10^1^	6.8 × 10^4^–6.8 × 10^0^
Tit/react ^4^	N/A	N/A	7.2 × 10^−3^	7.2 × 10^−4^	N/A	N/A	7.2 × 10^2^–7.2 × 10^−3^	7.2 × 10^0^–7.2/10^−4^
Tit/mL ^5^	N/A	N/A	5.6	0.56	N/A	N/A	5.6 × 10^5^–5.6 × 10^0^	5.6 × 10^3^–0.56

^1^ Dilution 10^x^; ^2^ Weight per reaction; ^3^ copies per reaction; ^4^ Titer (TCID_50_) per reaction; ^5^ Titer (TCID_50_) per milliliter.

**Table 3:** Dynamic range; Limit of Detection and Limit of Quantification with RG type VNNV.

	Limit of Detection				Dynamic Range for Detection			
	Plasmid		Crude Virus		Plasmid		Crude Virus	
	qPCR	ddPCR	qPCR	ddPCR	qPCR	ddPCR	qPCR	ddPCR
Dilution ^1^	−9	−10	−7	−9	−1 to −10	−5 to −11	−1 to −7	−3 to −9
w/reaction ^2^	46 ag	4.6 ag	0.26 fg	2.6 ag	4.6 ag–4.6 ng	0.46 pg–0.46 ag	0.26 fg–0.26 ng	2.6 ag–2.6 pg
cps/reaction ^3^	9.5	0.95	10.4	0.1	0.946–9.46 × 10^8^	9.46 × 10^−2^–9.46 × 10^4^	10.4–1.04 × 10^7^	0.1–1.04 × 10^5^
Tit/react ^4^	N/A	N/A	1.3 × 10^−3^	1.3 × 10^−5^	N/A	N/A	1.3 × 10^−3^–1.3 × 10^3^	1.3 × 10^−5^–1.3 × 10^1^
Tit/mL ^5^	N/A	N/A	1	1 × 10^−2^	N/A	N/A	1–1 × 10^6^	1 × 10^−2^–1 × 10^4^
	**Limit of Quantification**				**Dynamic range for quantification**			
	**Plasmid**		**Crude virus**		**Plasmid**		**Crude virus**	
	**qPCR**	**ddPCR**	**qPCR**	**ddPCR**	**qPCR**	**ddPCR**	**qPCR**	**ddPCR**
Dilution ^1^	−9	−9	−7	−7	−1 to −9	−5 to −9	−1 to −7	−3 to −7
w/reaction ^2^	46 ag	46 ag	0.26 fg	0.26 fg	4.6 ng–46 ag	0.46 pg–46 ag	0.26 ng–0.26 fg	2.6 pg–0.26 fg
cps/reaction ^3^	9.46	9.46	10.4	10.4	9.46 × 10^8^–9.46	9.46 × 10^4^–9.46	1.04 × 10^7^–10.4	1.04 × 10^5^–10.4
Tit/react ^4^	N/A	N/A	1.3 × 10^−3^	1.3 × 10^−3^	N/A	N/A	1.3 × 10^3^–1.3 × 10^−3^	1.3 × 10^1^–1.3 × 10^−3^
Tit/mL ^5^	N/A	N/A	1	1	N/A	N/A	1 × 10^6^–1	1 × 10^4^–1

^1^ Dilution 10^x^; ^2^ Weight per reaction; ^3^ copies per reaction; ^4^ Titer (TCID_50_) per reaction; ^5^ Titer (TCID_50_) per milliliter.

**Table 4:** Detection and quantification of VNNV genome copies in field samples (blood from *Solea senegallensis* breeders) by RT-qPCR and RT-ddPCR.

	RT-ddPCR	RT-qPCR	
Sample ID	Quantf ^1^	Ct	Quantf
273.22	8.0	≥40	0.0
274.22	1.4	39.5	1.3
275.22	ND	≥40	0.0
281.22	ND	≥40	0.0
282.22	27.8	≥40	0.0
283.22	14.3	≥40	0.0
290.22	ND	≥40	0.0
291.22	19.4	37.7	4.2
292.22	20.6	≥40	0.0
297.22	ND	39.7	1.1
298.22	4.4	35.1	23.5
299.22	5.2	≥40	0.0
305.22	ND	≥40	0.0
306.22	7.0	35.7	16.2
307.22	16.8	39.1	1.6
313.22	ND	≥40	0.0
314.22	7.8	38.9	1.8
315.22	ND	≥40	0.0
321.22	ND	39.8	1.0
322.22	4.3	≥40	0.0
323.22	0.0	≥40	0.0
329.22	0.0	≥40	0.0
330.22	0.0	≥40	0.0
331.22	0.0	≥40	0.0

Quantification (^1^) results (as copies/rctn) by RT-ddPCR and RT-qPCR are compared. For RT-qPCR, the threshold Ct (the maximum CT to consider a sample PCR positive) was originally 40 (Ct ≤ 40). In the second criteria, a threshold of 39.5 (Ct ≤ 39.5) was contemplated (data in red), and in those cases, the quantification was considered null.

**Table 5:** Reliability of the procedure was validated based on clinical parameters.

	A/Considering Positive Ct ≤ 40		B/Considering Positive Ct ≤ 39.5	
	Gold Standard: RT-qPCR		Gold Standard: RT-qPCR	
Test: RT-ddPCR	cSs = 0.75	PPV = 0.50	cSs = 1	PPV = 0.50
	cSp = 0.63	NPV = 0.83	cSp = 0.67	NPV = 1
	Gold Standard: RT-ddPCR		Gold Standard: RT-ddPCR	
Test: RT-qPCR	cSs = 0.5	PPV = 0.75	cSs = 0.5	PPV = 1.00
	cSp = 0.83	NPV = 0.63	cSp = 1	NPV = 0.67

cSs: clinical sensitivity; cSp: clinical specificity; PPV: positive predictive values; NPV: negative predictive value.

## Data Availability

Crude data will be available at “Dopazo, C.P., Bandín, I., Olveira, J.G., López-Vázquez, C., and Pereira, S.S. (29 July 2023). Droplet digital PCR (ddPCR) for diagnosis of VNNV. Retrieved from osf.io/ch4rn”.

## Supplementary Materials

The following supporting information can be downloaded at https://www.mdpi.com/article/10.3390/pathogens12091155/s1, Figure S1: Optimization of the primers and probe concentration: Total events and positive events. Figure S2: Reliability of the RT-ddPCR procedure for quantification of SJ crude virus. Results obtained from each repeat. Figure S3: Reliability of the ddPCR procedure for quantification of SJ pDNA. Results obtained from each repeat. Figure S4: Reliability of the RT-ddPCR procedure for quantification of RG crude virus. Results obtained from each repeat. Figure S5: Reliability of the ddPCR procedure for quantification of RG pDNA. Results obtained from each repeat. Table S1: Results obtained with ddPCR applied on SJ pDNA—Repeat 1. Table S2: Results obtained with ddPCR applied on SJ pDNA—Repeat 2. Table S3: Results obtained with ddPCR applied on SJ pDNA—Repeat 3. Table S4: Results obtained with ddPCR applied on SJ pDNA—Repeat 4. Table S5: Results obtained with ddPCR applied on SJ pDNA—Repeat 5. Table S6: Results obtained with ddPCR applied on RG pDNA—Repeat 1. Table S7: Results obtained with ddPCR applied on RG pDNA—Repeat 2. Table S8: Results obtained with ddPCR applied on SJ crude virus—Repeat 1. Table S9: Results obtained with ddPCR applied on SJ crude virus—Repeat 2. Table S10: Results obtained with ddPCR applied on RG crude virus—Repeat 1. Table S11: Results obtained with ddPCR applied on RG crude virus—Repeat 2. Table S12: Detection of SJNNV crude virus by RT-ddPCR and RT-qPCR. Table S13: Detection of SJNNV pDNA by ddPCR and qPCR. Table S14: Detection of RGNNV crude virus by RT-ddPCR and RT-qPCR. Table S15: Detection of RGNNV pDNA by ddPCR and qPCR.
