# Supplementary material for: Designing and Validation of a Droplet Digital PCR Procedure for Diagnosis and Accurate Quantification of Nervous Necrosis Virus in the Mediterranean Area
Source: Pathogens. 2023 Sep 12;12(9):1155. doi: 10.3390/pathogens12091155 (PMC10536565; doi:10.3390/pathogens12091155)
Supplement: Supplementary file 1 [file pathogens-12-01155-s001.zip › Supplementary Files/Suppl Tables/Suppl Table 12-SJ_CV all data-ddPCR y qPCR.pdf]

Supplementary Table 12.- Detection of SJNNV crude virus by RT-ddPCR and RT-qPCR

Concentration of the original sample					ddPCR (quantification of copies per reaction)								qPCR (quantification of copies per reaction)							
					Absolute data			Replicas[9]		Data in Lg_{10}			absolute data			Replicas		Data in Lg^{10}		
Dil[1]	Titer/ml[2]	Titer/react[3]	ng RNA/rctn[4]	cps/react[5]	Avrg[6]	StdDev[7]	CV[8]	nr	+	Avrg	StdDv	CV	Avrg[10]	Desv	CV	nr	+	Avrg	StdDv	CV
-1	5.6 x 10^{6}	7.2 x 10^{3}	0.18 ng	6.8 x 10^{7}	NT	NT	NT						NT	NT	NT					
-2	5.6 x 10^{5}	7.2 x 10^{2}	18 pg	6.8 x 10^{6}	NT	NT	NT						3.0 x 10^{6}	2.4 x 10^{6}	79.09	3	3	6.83	0.14	2.06
-3	5.6 x 10^{4}	7.2 x 10^{1}	1.8 pg	6.8 x 10^{5}	ND	ND	ND	3	0				3.6 x 10^{5}	3.2 x 10^{5}	89.89	3	3	5.90	0.16	2.68
-4	5.6 x 10^{3}	7.2 x 10^{0}	0.18 pg	6.8 x 10^{4}	7264.2	1815.6	25.0	6	6	3.85	0.11	2.76	3.0 x 10^{4}	2.1 x 10^{4}	70.65	3	3	4.83	0.14	2.93
-5	5.6 x 10^{2}	7.2 x 10^{-1}	18 fg	6.8 x 10^{3}	789.3	23.0	2.9	3	3	2.90	0.01	0.44	2.5 x 10^{3}	1.4 x 10^{3}	54.10	3	3	3.76	0.10	2.60
-6	5.6 x 10^{1}	7.2 x 10^{-2}	1.8 fg	6.8 x 10^{2}	98.0	17.1	17.4	3	3	1.99	0.08	3.80	3.3 x 10^{2}	5.6 x 10^{2}	167.94	3	3	2.68	0.59	22.01
-7	5.6 x 10^{0}	7.2 x 10^{-3}	0.18 fg	6.8 x 10^{1}	12.2	3.0	24.9	7	7	1.00	0.11	10.15	5.8 x 10^{1}	2.9 x 10^{1}	49.25	3	3	2.00	0.12	5.75
-8	5.6 x 10^{-1}	7.2 x 10^{-4}	18 ag	6.8 x 10^{0}	11.0	3.5	32.2	15	5	1.03	0.20	18.93	ND	ND	ND	3	0	-	-	-
-9	5.6 x 10^{-2}	7.2 x 10^{-5}	1.8 ag	6.8 x 10^{-1}	ND	ND	ND	15	0				NT	NT	NT					
-10	5.6 x 10^{-3}	7.2 x 10^{-6}	0.18 ag	6.8 x 10^{-2}	NT	NT	NT						NT	NT	NT					

1, Dilution; 2, Viral titer ($TCID_{50}$/ml) of crude virus (100µl were used for total RNA extraction); 3, Viral titer ($TCID_{50}$) per reaction (from the 70µl stock RNA, 9µl were used in the 20µl reverse transcription, and from this cDNA 2µl were employed in the 20µl PCR reaction); 4, corresponding ng of RNA used per PCR reaction; 5, number of genome copies per reaction (calculated from the formula γ=n/N x GL x ncMW described in M&M); 6, Average number of copies measured by RT-ddPCR from at least 3 replicas; 7, Standard Deviation; 8, Coefficient of Variation; 9, number of replicas used (nr) and number of replicas resulting positive PCR (+); 10, Average number of copies deduced from the equation y=-0.3067x+13.010 (Fig 4C). NT, Not tested; ND, Not detected.
