# Supplementary material for: Designing and Validation of a Droplet Digital PCR Procedure for Diagnosis and Accurate Quantification of Nervous Necrosis Virus in the Mediterranean Area
Source: Pathogens. 2023 Sep 12;12(9):1155. doi: 10.3390/pathogens12091155 (PMC10536565; doi:10.3390/pathogens12091155)
Supplement: Supplementary file 1 [file pathogens-12-01155-s001.zip › Supplementary Files/Suppl Tables/Suppl Table 13-SJ_dDNA all data-ddPCR y qPCR.pdf]

Supplementary Table 13.- Detection of SJNNV pDNA by ddPCR and qPCR

Concentration of the original sample			ddPCR (quantification of copies per reaction)								qPCR (quantification of copies per reaction)							
			Absolute data			Replicas[7]		Data in Lg_{10}			absolute data			Replicas		Data in Lg^{10}		
Dil[1]	ng pDNA/rctn[2]	cps/react[3]	Avrg[4]	StdDev[5]	CV[6]	nr	+	Avrg	StdDv	CV	Avrg[8]	Desv	CV	nr	+	Avrg	StdDv	CV
-1	9.0 ng	2.19×10^{9}	NT	NT	NT	NT	NT	NT	NT	NT	NT	NT	NT	NT	NT	NT	NT	NT
-2	0.9 ng	2.19×10^{8}	NT	NT	NT	NT	NT	NT	NT	NT	2.12E+09	2.05E+08	9.66	3	3	9.32	0.05	0.57
-3	90.0 pg	2.19×10^{7}	NT	NT	NT	NT	NT	NT	NT	NT	2.44E+08	1.30E+08	53.15	3	3	8.35	0.28	3.37
-4	9.0 pg	2.19×10^{6}	NT	NT	NT	NT	NT	NT	NT	NT	2.31E+07	5.07E+06	21.89	3	3	7.36	0.14	1.87
-5	0.9 pg	2.19×10^{5}	ND	ND	ND	11	0	ND	ND	ND	2.71E+06	5.70E+05	21.03	3	3	6.43	0.10	1.59
-6	90.0 fg	2.19×10^{4}	4352.7	1049.3	24.1	11	10	3.62	0.13	3.7	2.23E+05	7.33E+04	32.80	3	3	5.33	0.18	3.41
-7	9.0 fg	2.19×10^{3}	881.8	175.2	19.9	10	9	2.95	0.10	3.4	1.73E+04	2.95E+03	17.08	3	3	4.23	0.09	2.09
-8	0.9 fg	2.19×10^{2}	85.2	21.5	25.0	10	9	1.91	0.11	5.5	2.48E+03	3.02E+02	12.16	3	3	3.39	0.07	2.09
-9	90.0 ag	2.19×10^{1}	22.6	5.5	24.3	13	13	1.35	0.19	14.1	1.79E+02	9.31E+01	52.01	3	3	2.21	0.14	6.32
-10	9.0 ag	2.19×10^{0}	14.0	7.1	50.5	23	11	1.12	0.28	24.9	2.89E+01	3.36E+01	115.99	3	1	1.26	0.58	46.28
-11	0.9 ag	2.19×10^{-1}	26.8	21.5	80.2	23	2	1.34	0.40	29.4	ND	ND	ND	3	0	0.51	-	-
-12	0.09 ag	2.19×10^{-2}	ND	ND	ND	11	0	ND	ND	ND	ND	ND	ND	3	0	ND	ND	ND

1, Dilution; 2, pDNA concentration in ng/reaction; 3, pDNA copies per reaction (calculated from the formula γ=n/N x GL x ncMW described in M&M); 4, Average number of copies measured by ddPCR from at least 3 replicas; 5, Standard Deviation; 6, Coefficient of Variation; 7, number of replicas used (nr) and number of positive replicas (+); 8, Average number of copies deduced from the equation y=-0.3133x+12.533 (Fig 4D). NT, Not tested; ND, Not detected.
