# Supplementary material for: Designing and Validation of a Droplet Digital PCR Procedure for Diagnosis and Accurate Quantification of Nervous Necrosis Virus in the Mediterranean Area
Source: Pathogens. 2023 Sep 12;12(9):1155. doi: 10.3390/pathogens12091155 (PMC10536565; doi:10.3390/pathogens12091155)
Supplement: Supplementary file 1 [file pathogens-12-01155-s001.zip › Supplementary Files/Suppl Tables/Suppl Table 14-RG_CV all data-ddPCR y qPCR.pdf]

Supplementary Table 14.- Detection of RGNNV crude virus by RT-ddPCR and RT-qPCR

| Concentration of the original sample | | | | | ddPCR (quantification of copies per reaction) | | | | | | | | qPCR (quantification of copies per reaction) | | | | | | | |
|---|---|---|---|---|---|---|---|---|---|---|---|---|---|---|---|---|---|---|---|---|
| | | | | | Absolute data | | | Replicas[9] | | Data in Lg[10] | | | absolute data | | | Replicas | | Data in Lg[10] | | |
| Dil[1] | Titer/ml[2] | Titer/react[3] | ngRNA/rctn[4] | cps/react[5] | Avrg[6] | StdDev[7] | CV[8] | nr | + | Avrg | StdDv | CV | Avrg[10] | Desv | CV | nr | + | Avrg | StdDv | CV |
| -1 | $1 \times 10^{6}$ | $1.3 \times 10^{3}$ | 0.26 ng | $1.04 \times 10^{7}$ | NT | NT | NT | 3 | 0 | - | - | - | $6.5 \times 10^{7}$ | $1.1 \times 10^{7}$ | 10.65 | 3 | 3 | 7.99 | 0.05 | 0.59 |
| -2 | $1 \times 10^{5}$ | $1.3 \times 10^{2}$ | 26 pg | $1.04 \times 10^{6}$ | ND | ND | ND | 3 | 0 | ND | - | - | $5.7 \times 10^{6}$ | $1.2 \times 10^{6}$ | 13.89 | 3 | 3 | 6.94 | 0.06 | 0.89 |
| -3 | $1 \times 10^{4}$ | $1.3 \times 10^{1}$ | 2.6 pg | $1.04 \times 10^{5}$ | 24946.7 | 2412.5 | 9.7 | 3 | 3 | 4.4 | 0.0 | 1.0 | $8.6 \times 10^{5}$ | $1.8 \times 10^{5}$ | 13.36 | 3 | 3 | 6.11 | 0.06 | 0.94 |
| -4 | $1 \times 10^{3}$ | $1.3 \times 10^{0}$ | 0.26 pg | $1.04 \times 10^{4}$ | 2880.0 | 348.7 | 12.1 | 3 | 3 | 3.5 | 0.1 | 1.5 | $7.1 \times 10^{4}$ | $1.4 \times 10^{4}$ | 13.23 | 3 | 3 | 5.03 | 0.06 | 1.14 |
| -5 | $1 \times 10^{2}$ | $1.3 \times 10^{-1}$ | 26 fg | $1.04 \times 10^{3}$ | 199.3 | 42.4 | 21.3 | 3 | 3 | 2.3 | 0.1 | 4.3 | $7.5 \times 10^{3}$ | $1.0 \times 10^{3}$ | 9.14 | 3 | 3 | 4.05 | 0.04 | 0.98 |
| -6 | $1 \times 10^{1}$ | $1.3 \times 10^{-2}$ | 2.6 fg | $1.04 \times 10^{2}$ | 31.3 | 4.2 | 13.3 | 3 | 3 | 1.5 | 0.1 | 3.8 | $7.1 \times 10^{2}$ | $1.2 \times 10^{2}$ | 10.66 | 3 | 3 | 3.03 | 0.05 | 1.56 |
| -7 | $1 \times 10^{0}$ | $1.3 \times 10^{-3}$ | 0.26 fg | $1.04 \times 10^{1}$ | 6.3 | 1.3 | 21.0 | 11 | 7 | 0.8 | 0.1 | 11.4 | $6.1 \times 10^{1}$ | $2.3 \times 10^{1}$ | 24.76 | 3 | 3 | 1.96 | 0.11 | 5.36 |
| -8 | $1 \times 10^{-1}$ | $1.3 \times 10^{-4}$ | 26 a | $1.04 \times 10^{0}$ | 19.3 | 20.5 | 106.0 | 11 | 4 | 1.5 | 0.6 | 40.9 | ND | ND | ND | | | - | - | - |
| -9 | $1 \times 10^{-2}$ | $1.3 \times 10^{-5}$ | 2.6 ag | $1.04 \times 10^{-1}$ | 18.7 | 4.5 | 24.0 | 11 | 4 | 1.3 | 0.1 | 9.0 | NT | NT | NT | | | | | |
| -10 | $1 \times 10^{-3}$ | $1.3 \times 10^{-6}$ | 0.26 ag | $1.04 \times 10^{-2}$ | NT | NT | NT | | | | | | NT | NT | NT | | | | | |

1, Dilution; 2, Viral titer ($TCID_{50}$/ml) of crude virus (100µl were used for total RNA extraction); 3, Viral titer ($TCID_{50}$) per reaction (from the 70µl stock RNA, 9µl were used in the 20µl reverse transcription, and from this cDNA 2µl were employed in the 20µl PCR reaction); 4, corresponding ng of RNA used per PCR reaction; 5, number of genome copies per reaction (calculated from the formula $\gamma$=n/N x GL x ncMW described in M&M); 6, Average number of copies measured by RT-ddPCR from at least 3 replicas; 7, Standard Deviation; 8, Coefficient of Variation; 9, number of replicas used (nr) and number of replicas resulting positive PCR (+); 10, Average number of copies deduced from the equation y=-0.2932x+12.544 (Fig 5C). NT, Not tested; ND, Not detected.
