# Supplementary material for: Designing and Validation of a Droplet Digital PCR Procedure for Diagnosis and Accurate Quantification of Nervous Necrosis Virus in the Mediterranean Area
Source: Pathogens. 2023 Sep 12;12(9):1155. doi: 10.3390/pathogens12091155 (PMC10536565; doi:10.3390/pathogens12091155)
Supplement: Supplementary file 1 [file pathogens-12-01155-s001.zip › Supplementary Files/Suppl Tables/Suppl Table 15-RG_pDNA all data-ddPCR y qPCR.pdf]

Supplementary Table 15.- Detection of RGNNV pDNA by ddPCR and qPCR

Concentration of the original sample			ddPCR (quantification of copies per reaction)								qPCR (quantification of copies per reaction)							
			Absolute data			Replicas[7]		Data in Lg10			absolute data			Replicas		Data in Lg10		
Dil[1]	ng pDNA/rctn[2]	cps/react[3]	Avrg[4]	StdDev[5]	CV[6]	nr	+	Avrg[8]	StdDv	CV	Avrg[5]	Desv	CV	nr	+	Avrg	StdDv	CV
-1	4.60 ng	9.46×10^{8}	NT	NT	NT	0	0	NT	NT	NT	6.5×10^{8}	1.5×10^{8}	23.11	NT	NT	8.81	1.00	1.1
-2	0.46 ng	9.46×10^{7}	NT	NT	NT	0	0	NT	NT	NT	1.2×10^{8}	1.3×10^{7}	10.81	3	3	8.07	0.05	0.6
-3	46.00 pg	9.46×10^{6}	NT	NT	NT	0	0	NT	NT	NT	1.3×10^{7}	1.1×10^{6}	8.51	3	3	7.10	0.04	0.5
-4	4.60 pg	9.46×10^{5}	ND	ND	ND	3	0	ND	ND	ND	8.6×10^{5}	8.4×10^{4}	9.69	3	3	5.93	0.04	0.7
-5	0.46 pg	9.46×10^{4}	14166.7	3209.4	22.7	3	3	4.15	0.1	2.5	7.6×10^{4}	9.2×10^{3}	12.05	3	3	4.88	0.05	1.0
-6	46.00 fg	9.46×10^{3}	1760.0	174.4	9.92	3	3	3.25	0.0	1.4	1.1×10^{4}	1.7×10^{3}	15.00	3	3	4.05	0.07	1.6
-7	4.60 fg	9.46×10^{2}	238.7	24.2	10.1	3	3	2.38	0.0	1.8	9.7×10^{2}	1.2×10^{2}	12.00	3	3	2.98	0.05	1.7
-8	0.46 fg	9.46×10^{1}	28.7	4.6	16.1	3	3	1.46	0.1	4.6	1.3×10^{2}	2.0×10^{1}	15.36	3	3	2.11	0.06	3.1
-9	46.00 ag	9.46×10^{0}	16.3	3.6	21.7	3	2	1.21	0.0	3.7	9.7×10^{0}	2.2×10^{0}	22.35	3	3	0.98	0.10	10.5
-10	4.60 ag	9.46×10^{-1}	13.5	4.0	29.5	11	8	1.13	0.1	12.4	ND	-	-	3	1	-0.14		
-11	0.46 ag	9.46×10^{-2}	18.0			11	2	1.26	-	-	NT	NT	NT					

1, Dilution; 2, pDNA concentration in ng/reaction; 3, pDNA copies per reaction (calculated from the formula γ=n/N x GL x ncMW described in M&M); 4, Average number of copies measured by ddPCR from at least 3 replicas; 5, Standard Deviation; 6, Coefficient of Variation; 7, number of replicas used (nr) and number of positive replicas (+); 8, Average number of copies deduced from the equation y=-0.290x+11.547 (Fig 5D). NT, Not tested; ND, Not detected.
