# Supplementary material for: Designing and Validation of a Droplet Digital PCR Procedure for Diagnosis and Accurate Quantification of Nervous Necrosis Virus in the Mediterranean Area
Source: Pathogens. 2023 Sep 12;12(9):1155. doi: 10.3390/pathogens12091155 (PMC10536565; doi:10.3390/pathogens12091155)
Supplement: Supplementary file 1 [file pathogens-12-01155-s001.zip › Supplementary Files/Suppl Tables/Suppl Tables 10_11- Data-ddPCR-RG-CV-Repts1_2.pdf]

Supplementary Table 10.- Results obtained with ddPCR applied on RG crude virus – Repeat 1

Dil[1]	Titer[2]	[RNA] ng/µl[3]		Titer/react[4]	ng RNA/rctn[5]	cps/react[6]	Replica[7]					
							1	2	3	Avrg[8]	Desv[9]	CV[10]
-2	1×10^{5}	2×10^{-2}	(20.0 pg)	1.3×10^{2}	2.57×10^{-2}	1.04×10^{6}	ND	ND	ND	ND	ND	ND
-3	1×10^{4}	2×10^{-3}	(2.0 pg)	1.3×10^{1}	2.57×10^{-3}	1.04×10^{5}	27420	22600	24820	24946.7	2412.5	9.67
-4	1×10^{3}	2×10^{-4}	(0.2 pg)	1.3×10^{0}	2.57×10^{-4}	1.04×10^{4}	2640	3280	2720	2880.0	348.7	12.11
-5	1×10^{2}	2×10^{-5}	(20.0 fg)	1.3×10^{-1}	2.57×10^{-5}	1.04×10^{3}	152	212	234	199.3	42.4	21.29
-6	1×10^{1}	2×10^{-6}	(2.0 fg)	1.3×10^{-2}	2.57×10^{-6}	1.04×10^{2}	28	30	36	31.3	4.2	13.29
-7	1×10^{0}	2×10^{-7}	(0.2 fg)	1.3×10^{-3}	2.57×10^{-7}	1.04×10^{1}	ND	7.6	ND	7.6	0.0	0.00
-8	1×10^{-1}	2×10^{-8}	(20.0 ag)	1.3×10^{-4}	2.57×10^{-8}	1.04×10^{0}	9	ND	ND	9	-	-
-9	1×10^{-2}	2×10^{-9}	(2.0 ag)	1.3×10^{-5}	2.57×10^{-9}	1.04×10^{-1}	22	12.6	ND	17.3	6.65	38.42
-10	1×10^{-3}	2×10^{-10}	(0.2 ag)	1.3×10^{-6}	2.57×10^{-10}	1.04×10^{-2}	NT	NT	NT	NT	NT	NT

1, Dilution; 2, Viral titer ($TCID_{50}$/ml) of crude virus (100µl were used for total RNA extraction); 3, RNA concentration in ng/µl of crude virus; 4, Viral titer ($TCID_{50}$) per reaction (from the 70µl stock RNA, 9µl were used in the 20µl reverse transcription, and from this cDNA 2µl were employed in the 20µl PCR reaction); 5, corresponding ng of RNA used per PCR reaction; 6, number of genome copies per reaction (calculated from the formula γ=n/N x GL x ncMW described in M&M); 7, Number of genome copies per reaction measured by RT-ddPCR from 3 replicas 8, Average number of copies; 9, Standard Deviation; 10, Coefficient of Variation. NT, Not tested; ND, Not detected

Supplementary Table 10.- Results obtained with ddPCR applied on RG crude virus – Repeat 2

Dil[1]	Titer[2]	[RNA] ng/µl[3]		Titer/react[4]	ng RNA/rctn[5]	cps/react[6]	Replica[7]										
							1	2	3	4	5	6	7	8	Avrg[8]	Desv[9]	CV[10]
-7	1 x 10^{0}	2 x 10^{-7}	(0.2 fg)	1.3 x 10^{-3}	2.57 x 10^{-7}	1.04 x 10^{1}	ND	4.8	6.6	ND	5.2	6.4	8.3	5.2	6.1	1.3	21.4
-8	1 x 10^{-1}	2 x 10^{-8}	(20.0 ag)	1.3 x 10^{-4}	2.57 x 10^{-8}	1.04 x 10^{0}	ND	11	ND	7.2	ND	ND	ND	50	22.7	23.7	104
-9	1 x 10^{-2}	2 x 10^{-9}	(2.0 ag)	1.3 x 10^{-5}	2.57 x 10^{-9}	1.04 x 10^{-1}	ND	ND	ND	18	ND	ND	22	ND	20.0	2.8	14.1

1, Dilution; 2, Viral titer ($TCID_{50}$/ml) of crude virus (100µl were used for total RNA extraction); 3, RNA concentration in ng/µl of crude virus; 4, Viral titer ($TCID_{50}$) per reaction (from the 70µl stock RNA, 9µl were used in the 20µl reverse transcription, and from this cDNA 2µl were employed in the 20µl PCR reaction); 5, corresponding ng of RNA used per PCR reaction; 6, number of genome copies per reaction (calculated from the formula γ=n/N x GL x ncMW described in M&M); 7, Number of genome copies per reaction measured by RT-ddPCR from 3 replicas 8, Average number of copies; 9, Standard Deviation; 10, Coefficient of Variation. NT, Not tested; ND, Not detected
