# Supplementary material for: Designing and Validation of a Droplet Digital PCR Procedure for Diagnosis and Accurate Quantification of Nervous Necrosis Virus in the Mediterranean Area
Source: Pathogens. 2023 Sep 12;12(9):1155. doi: 10.3390/pathogens12091155 (PMC10536565; doi:10.3390/pathogens12091155)
Supplement: Supplementary file 1 [file pathogens-12-01155-s001.zip › Supplementary Files/Suppl Tables/Suppl Tables 1_5- Data-ddPCR-SJ-pDNA-Repts1_5.pdf]

Supplementary Table 1.- Results obtained with ddPCR applied on SJ pDNA – Repeat 1

					Replica[5]					
Dil[1]	ng/µl[2]	ng pDNA/rctn[3]		cps/react[4]	1	2	3	Avrg[6]	Dev[7]	CV[8]
-4	4.5×10^{-3}	9.0×10^{-3}	(9.0 pg)	2.19×10^{6}	NT	NT	NT	ND	ND	ND
-5	4.5×10^{-4}	9.0×10^{-4}	(0.90 pg)	2.19×10^{5}	ND	ND	ND	-	-	-
-6	4.5×10^{-5}	9.0×10^{-5}	(90.0 fg)	2.19×10^{4}	4720	4700	ND	4710.0	14.14	0.3
-7	4.5×10^{-6}	9.0×10^{-6}	(9.0 fg)	2.19×10^{3}	718	1060	ND	889.0	221.83	24.95
-8	4.5×10^{-7}	9.0×10^{-7}	(0.90 fg)	2.19×10^{2}	108.0	108.0	120.0	112.0	6.92	6.19
-9	4.5×10^{-8}	9.0×10^{-8}	(90.0 ag)	2.19×10^{1}	30.0	20.0	20.0	23.3	5.78	24.74
-10	4.5×10^{-9}	9.0×10^{-9}	(9.0 ag)	2.19×10^{0}	16.0	ND	ND	16.0	-	-
-11	4.5×10^{-10}	9.0×10^{-10}	(0.90 ag)	2.19×10^{-1}	11.6	ND	ND	11.6	-	-
-12	4.5×10^{-11}	9.0×10^{-11}	(0.09 ag)	2.19×10^{-2}	ND	ND	ND	-	-	-

1, Dilution; 2, Concentration pf plasmid DNA tested; 3, pDNA concentration per reaction; 4, number of genome copies per reaction (calculated from the formula γ=n/N x GL x ncMW described in M&M); 5, Number of pDNA copies per reaction measured by ddPCR from at least 3 replicas; 6, Average number of copies; 7, Standard Deviation; 8, Coefficient of Variation. NT, Not tested; ND, Not detected

Supplementary Table 2.- Results obtained with ddPCR applied on SJ pDNA – Repeat 2

					Replica[5]					
Dil[1]	ng/µl[2]	ng pDNA/rctn[3]		cps/react[4]	1	2	3	Avrg[6]	Desv[7]	CV[8]
-4	4.5×10^{-3}	9.0×10^{-3}	(9.0 pg)	2.19×10^{6}	NT	NT	NT	-	-	-
-5	4.5×10^{-4}	9.0×10^{-4}	(0.90 pg)	2.19×10^{5}	ND	ND	ND	ND	-	-
-6	4.5×10^{-5}	9.0×10^{-5}	(90.0 fg)	2.19×10^{4}	5320	5180	5160	5220.0	87.18	1.67
-7	4.5×10^{-6}	9.0×10^{-6}	(9.0 fg)	2.19×10^{3}	1006	1074	1008	1029.3	38.70	3.76
-8	4.5×10^{-7}	9.0×10^{-7}	(0.90 fg)	2.19×10^{2}	78.0	72.0	90.0	80.0	9.17	11.46
-9	4.5×10^{-8}	9.0×10^{-8}	(90.0 ag)	2.19×10^{1}	19.3	15.0	14.9	16.4	2.5	15.3
-10	4.5×10^{-9}	9.0×10^{-9}	(9.0 ag)	2.19×10^{0}	ND	13.2	20.0	16.6	4.81	28.97
-11	4.5×10^{-10}	9.0×10^{-10}	(0.90 ag)	2.19×10^{-1}	ND	ND	ND	ND	-	-
-12	4.5×10^{-11}	9.0×10^{-11}	(0.09 ag)	2.19×10^{-2}	NT	NT	NT	-	-	-

1, Dilution; 2, Concentration pf plasmid DNA tested; 3, pDNA concentration per reaction; 4, number of genome copies per reaction (calculated from the formula γ=n/N x GL x ncMW described in M&M); 5, Number of pDNA copies per reaction measured by ddPCR from at least 3 replicas; 6, Average number of copies; 7, Standard Deviation; 8, Coefficient of Variation. NT, Not tested; ND, Not detected

Supplementary Table 3.- Results obtained with ddPCR applied on SJ pDNA – Repeat 3

Dil[1]	ng/µl[2]	ng pDNA/rctn[3]		cps/react[4]	Replica[5]			Avrg[6]	Desv[7]	CV[8]
					1	2	3			
-4	4.5 x 10^{-3}	9.0 x 10^{-3}	(9.0 pg)	2.19 x 10^{6}	NT	NT	NT	-	-	-
-5	4.5 x 10^{-4}	9.0 x 10^{-4}	(0.90 pg)	2.19 x 10^{5}	ND	ND	ND	ND	-	-
-6	4.5 x 10^{-5}	9.0 x 10^{-5}	(90.0 fg)	2.19 x 10^{4}	4138	4838	4351	4442.3	358.83	8.08
-7	4.5 x 10^{-6}	9.0 x 10^{-6}	(9.0 fg)	2.19 x 10^{3}	566.0	908	844.0	772.7	181.82	23.53
-8	4.5 x 10^{-7}	9.0 x 10^{-7}	(0.90 fg)	2.19 x 10^{2}	58.0	56.0	84.2	66.1	15.74	23.82
-9	4.5 x 10^{-8}	9.0 x 10^{-8}	(90.0 ag)	2.19 x 10^{1}	24.0	16.0	20.0	20.0	4.00	20.00
-10	4.5 x 10^{-9}	9.0 x 10^{-9}	(9.0 ag)	2.19 x 10^{0}	ND	ND	11.4	14.4	-	-
-11	4.5 x 10^{-10}	9.0 x 10^{-10}	(0.90 ag)	2.19 x 10^{-1}	ND	ND	ND	ND	-	-
-12	4.5 x 10^{-11}	9.0 x 10^{-11}	(0.09 ag)	2.19 x 10^{-2}	NT	NT	NT	-	-	-

1, Dilution; 2, Concentration pf plasmid DNA tested; 3, pDNA concentration per reaction; 4, number of genome copies per reaction (calculated from the formula γ=n/N x GL x ncMW described in M&M); 5, Number of pDNA copies per reaction measured by ddPCR from at least 3 replicas; 6, Average number of copies; 7, Standard Deviation; 8, Coefficient of Variation. NT, Not tested; ND, Not detected

Supplementary Table 4.- Results obtained with ddPCR applied on SJ pDNA – Repeat 4

Dil[1]	ng/µl[2]	ng pDNA/rctn[3]		cps/react[4]	Replica[5]			Avrg[6]	Desv[7]	CV[8]
					1	2	3			
-4	4.5 x 10^{-3}	9.0 x 10^{-3}	(9.0 pg)	2.19 x 10^{6}	NT	NT	NT	-	-	-
-5	4.5 x 10^{-4}	9.0 x 10^{-4}	(0.90 pg)	2.19 x 10^{5}	ND	NT	NT	ND	-	-
-6	4.5 x 10^{-5}	9.0 x 10^{-5}	(90.0 fg)	2.19 x 10^{4}	3140	NT	NT	3140	-	-
-7	4.5 x 10^{-6}	9.0 x 10^{-6}	(9.0 fg)	2.19 x 10^{3}	752	NT	NT	752	-	-
-8	4.5 x 10^{-7}	9.0 x 10^{-7}	(0.90 fg)	2.19 x 10^{2}	78	NT	NT	78	-	-
-9	4.5 x 10^{-8}	9.0 x 10^{-8}	(90.0 ag)	2.19 x 10^{1}	22	NT	NT	22	-	-
-10	4.5 x 10^{-9}	9.0 x 10^{-9}	(9.0 ag)	2.19 x 10^{0}	9.8	NT	NT	9.8	-	-
-11	4.5 x 10^{-10}	9.0 x 10^{-10}	(0.90 ag)	2.19 x 10^{-1}	ND	NT	NT	ND	-	-
-12	4.5 x 10^{-11}	9.0 x 10^{-11}	(0.09 ag)	2.19 x 10^{-2}	ND	NT	NT	ND	-	-

1, Dilution; 2, Concentration pf plasmid DNA tested; 3, pDNA concentration per reaction; 4, number of genome copies per reaction (calculated from the formula γ=n/N x GL x ncMW described in M&M); 5, Number of pDNA copies per reaction measured by ddPCR from at least 3 replicas; 6, Average number of copies; 7, Standard Deviation; 8, Coefficient of Variation. NT, Not tested; ND, Not detected

Supplementary Table 5.- Results obtained with ddPCR applied on SJ pDNA – Repeat 5

Dil[1]	ng/µl[2]	ng RNA/rctn[3]	cps/react[4]	Replica[5]														
				1	2	3	4	5	6	7	8	9	10	11	12	Avrg[6]	Desv[7]	CV[8]
-5	4.5×10^{-4}	9.0×10^{-4}	2.19×10^{5}	NT	NT	NT	NT	NT	NT	NT	NT	NT	NT	NT	NT	NT	-	-
-6	4.5×10^{-5}	9.0×10^{-5}	2.19×10^{4}	1980	NT	NT	NT	NT	NT	NT	NT	NT	NT	NT	NT	1980	-	-
-7	4.5×10^{-6}	9.0×10^{-6}	2.19×10^{3}	NT	NT	NT	NT	NT	NT	NT	NT	NT	NT	NT	NT	NT	-	-
-8	4.5×10^{-7}	9.0×10^{-7}	2.19×10^{2}	NT	NT	NT	NT	NT	NT	NT	NT	NT	NT	NT	NT	NT	-	-
-9	4.5×10^{-8}	9.0×10^{-8}	2.19×10^{1}	30.0	31.0	32.0	NT	NT	NT	NT	NT	NT	NT	NT	NT	31.0	1.0	3.2
-10	4.5×10^{-9}	9.0×10^{-9}	2.19×10^{0}	ND	ND	6.0	ND	12.8	4.8	24	ND	ND	26.9	9.4	ND	14.0	9.4	66.9
-11	4.5×10^{-10}	9.0×10^{-10}	2.19×10^{-1}	ND	ND	ND	ND	ND	42	ND	ND	ND	ND	ND	ND	42.0	-	-
-12	4.5×10^{-11}	9.0×10^{-11}	2.19×10^{-2}	ND	NT	NT	NT	NT	NT	NT	NT	NT	NT	NT	NT	NT	-	-

1, Dilution; 2, Concentration pf plasmid DNA tested; 3, pDNA concentration per reaction; 4, number of genome copies per reaction (calculated from the formula γ=n/N x GL x ncMW described in M&M); 5, Number of pDNA copies per reaction measured by ddPCR from at least 3 replicas; 6, Average number of copies; 7, Standard Deviation; 8, Coefficient of Variation. NT, Not tested; ND, Not detected
