# Supplementary material for: Designing and Validation of a Droplet Digital PCR Procedure for Diagnosis and Accurate Quantification of Nervous Necrosis Virus in the Mediterranean Area
Source: Pathogens. 2023 Sep 12;12(9):1155. doi: 10.3390/pathogens12091155 (PMC10536565; doi:10.3390/pathogens12091155)
Supplement: Supplementary file 1 [file pathogens-12-01155-s001.zip › Supplementary Files/Suppl Tables/Suppl Tables 6_7- Data-ddPCR-RG-pDNA-Repts1_2.pdf]

Supplementary Table 6.- Results obtained with ddPCR applied on RG pDNA – Repeat 1

Dil[1]	ng/µl[2]	ng pDNA/rctn[3]		cps/react[4]	Replica[5]			Avrg[6]	Desv[7]	CV[8]
					1	2	3			
-4	2.3×10^{-3}	4.6×10^{-3}	(4.6 pg)	9.46×10^{5}	ND	ND	ND	ND	ND	ND
-5	2.3×10^{-4}	4.6×10^{-4}	(0.46 pg)	9.46×10^{4}	10540	15320	16640	14166,7	3209.4	22,65
-6	2.3×10^{-5}	4.6×10^{-5}	(46.0 fg)	9.46×10^{3}	1560	1880	1840	1760.0	174.4	9.91
-7	2.3×10^{-6}	4.6×10^{-6}	(4.6 fg)	9.46×10^{2}	220	230	266	238.7	24.2	10.14
-8	2.3×10^{-7}	4.6×10^{-7}	(0.46 fg)	9.46×10^{1}	26	34	26	28.7	4.6	16.11
-9	2.3×10^{-8}	4.6×10^{-8}	(46.0 ag)	9.46×10^{0}	19.5	12.5	17	16.3	3.55	21.72
-10	2.3×10^{-9}	4.6×10^{-9}	(4.6 ag)	9.46×10^{-1}	ND	18	ND	18,0	-	-
-11	2.3×10^{-10}	4.6×10^{-10}	(0.46 ag)	9.46×10^{-2}	NT	NT	NT	NT	NT	NT

1, Dilution; 2, Concentration pf plasmid DNA tested; 3, pDNA concentration per reaction; 4, number of genome copies per reaction (calculated from the formula γ=n/N x GL x ncMW described in M&M); 5, Number of pDNA copies per reaction measured by ddPCR from at least 3 replicas; 6, Average number of copies; 7, Standard Deviation; 8, Coefficient of Variation. NT, Not tested; ND, Not detected

Supplementary Table 7.- Results obtained with ddPCR applied on RG pDNA – Repeat 2

Dil[1]	ng/µl[2]	ng pDNA/rctn[3]	cps/react[4]	Replica[5]										
				1	2	3	4	5	6	7	8	Avrg[6]	Desv[7]	CV[8]
-10	2.3×10^{-9}	4.60×10^{-9}	9.46×10^{-1}	12.6	10.8	7.4	ND	15.0	9.8	14.0	16.0	12.2	3.1	25.0
-11	2.3×10^{-10}	4.60×10^{-10}	9.46×10^{-2}	ND	ND	ND	ND	ND	ND	ND	18.0	18.0		

1, Dilution; 2, Concentration pf plasmid DNA tested; 3, pDNA concentration per reaction; 4, number of genome copies per reaction (calculated from the formula γ=n/N x GL x ncMW described in M&M); 5, Number of pDNA copies per reaction measured by ddPCR from at least 3 replicas; 6, Average number of copies; 7, Standard Deviation; 8, Coefficient of Variation. NT, Not tested; ND, Not detected
