# Supplementary material for: Designing and Validation of a Droplet Digital PCR Procedure for Diagnosis and Accurate Quantification of Nervous Necrosis Virus in the Mediterranean Area
Source: Pathogens. 2023 Sep 12;12(9):1155. doi: 10.3390/pathogens12091155 (PMC10536565; doi:10.3390/pathogens12091155)
Supplement: Supplementary file 1 [file pathogens-12-01155-s001.zip › Supplementary Files/Suppl Tables/Suppl Tables 8_9- Data-ddPCR-SJ-CV-Repts1_2.pdf]

Supplementary Table 8.- Results obtained with ddPCR applied on SJ crude virus – Repeat 1

Dil[1]	Titer[2]	[RNA] ng/µl[3]		Titer/react[4]	ng RNA/rctn[5]	cps/react[6]	Replica[7]					
							1	2	3	Avrg[8]	Desv[9]	CV[10]
-2	5.6×10^{5}	2×10^{-2}	(20.0 pg)	7.2×10^{2}	1.8×10^{-2}	6.8×10^{6}	NT	NT	NT	NT		
-3	5.6×10^{4}	2×10^{-3}	(2.0 pg)	7.2×10^{1}	1.8×10^{-3}	6.8×10^{5}	ND	ND	ND	ND		
-4	5.6×10^{3}	2×10^{-4}	(0.2 pg)	7.2×10^{0}	1.8×10^{-4}	6.8×10^{4}	9660	7380	9240	8760	1213.4	13.9
-5	5.6×10^{2}	2×10^{-5}	(20.0 fg)	7.2×10^{-1}	1.8×10^{-5}	6.8×10^{3}	766	812	790	789.3	23.0	2.9
-6	5.6×10^{1}	2×10^{-6}	(2.0 fg)	7.2×10^{-2}	1.8×10^{-6}	6.8×10^{2}	82	116	96	98.0	17.1	17.4
-7	5.6×10^{0}	2×10^{-7}	(0.2 fg)	7.2×10^{-3}	1.8×10^{-7}	6.8×10^{1}	13,2	10	8.2	10.5	2.5	24.2
-8	5.6×10^{-1}	2×10^{-8}	(20.0 ag)	7.2×10^{-4}	1.8×10^{-8}	6.8×10^{0}	16	ND	ND	16.0		
-9	5.6×10^{-2}	2×10^{-9}	(2.0 ag)	7.2×10^{-5}	1.8×10^{-9}	6.8×10^{-1}	ND	ND	ND	ND		
-10	5.6×10^{-3}	2×10^{-10}	(0.2 ag)	7.2×10^{-6}	1.8×10^{-10}	6.8×10^{-2}	ND	ND	ND	ND		

1, Dilution; 2, Viral titer ($TCID_{50}$/ml) of crude virus (100µl were used for total RNA extraction); 3, RNA concentration in ng/µl of crude virus; 4, Viral titer ($TCID_{50}$) per reaction (from the 70µl stock RNA, 9µl were used in the 20µl reverse transcription, and from this cDNA 2µl were employed in the 20µl PCR reaction); 5, corresponding ng of RNA used per PCR reaction; 6, number of genome copies per reaction (calculated from the formula $\gamma = n/N \times GL \times ncMW$ described in M&M); 7, Number of genome copies per reaction measured by RT-ddPCR from 3 replicas 8, Average number of copies; 9, Standard Deviation; 10, Coefficient of Variation. NT, Not tested; ND, Not detected

Supplementary Table 9.- Results obtained with ddPCR applied on SJ crude virus – Repeat 2

							Replica[7]														
Dil[1]	Titer[2]	[RNA] ng/µl[3]		Titer/rctn[4]	ng RNA/rctn[5]	cps/react[6]	1	2	3	4	5	6	7	8	9	10	11	12	Avrg[8]	Desv[9]	CV[10]
-3	5.6×10^{4}	2×10^{-3}	(2.0 pg)	7.2×10^{1}	1.8×10^{-3}	6.8×10^{5}	NT	NT	NT	NT	NT	NT	NT	NT	NT	NT	NT	NT	-	-	-
-4	5.6×10^{3}	2×10^{-4}	(0.2 pg)	7.2×10^{0}	1.8×10^{-4}	6.8×10^{4}	6020	5735	5550	NT	NT	NT	NT	NT	NT	NT	NT	NT	5768.3	236.8	4.1
-5	5.6×10^{2}	2×10^{-5}	(20.0 fg)	7.2×10^{-1}	1.8×10^{-5}	6.8×10^{3}	NT	NT	NT	NT	NT	NT	NT	NT	NT	NT	NT	NT	-	-	-
-6	5.6×10^{1}	2×10^{-6}	(2.0 fg)	7.2×10^{-2}	1.8×10^{-6}	6.8×10^{2}	NT	NT	NT	NT	NT	NT	NT	NT	NT	NT	NT	NT	-	-	-
-7	5.6×10^{0}	2×10^{-7}	(0.2 fg)	7.2×10^{-3}	1.8×10^{-7}	6.8×10^{1}	12.3	16.6	13.4	9.8	NT	NT	NT	NT	NT	NT	NT	NT	13.0	2,8	21.6
-8	5.6×10^{-1}	2×10^{-8}	(20.0 ag)	7.2×10^{-4}	1.8×10^{-8}	6.8×10^{0}	ND	13.3	10.8	ND	ND	ND	ND	8.8	5.8	11.1	ND	ND	10.0	2.8	28.3
-9	5.6×10^{-2}	2×10^{-9}	(2.0 ag)	7.2×10^{-5}	1.8×10^{-9}	6.8×10^{-1}	ND	ND	ND	ND	ND	ND	ND	ND	ND	ND	ND	ND	-	-	-
-10	5.6×10^{-3}	2×10^{-9}	(2.0 ag)	7.2×10^{-6}	1.8×10^{-10}	6.8×10^{-2}	NT	NT	NT	NT	NT	NT	NT	NT	NT	NT	NT	NT			

1, Dilution; 2, Viral titer ($TCID_{50}$/ml) of crude virus (100µl were used for total RNA extraction); 3, RNA concentration in ng/µl of crude virus; 4, Viral titer ($TCID_{50}$) per reaction (from the 70µl stock RNA, 9µl were used in the 20µl reverse transcription, and from this cDNA 2µl were employed in the 20µl PCR reaction); 5, corresponding ng of RNA used per PCR reaction; 6, number of genome copies per reaction (calculated from the formula γ=n/N x GL x ncMW described in M&M); 7, Number of genome copies per reaction measured by RT-ddPCR from 3 replicas 8, Average number of copies; 9, Standard Deviation; 10, Coefficient of Variation. NT, Not tested; ND, Not detected
